# Introduction to the Special Issue on Image-Based Information Retrieval from the Web

**DOI:** 10.3390/jimaging5070062

**Published:** 2019-06-30

**Authors:** Phivos Mylonas, Evaggelos Spyrou

**Affiliations:** 1 Department of Informatics, Ionian University, Corfu 49132, Greece; 2 General Department, University of Thessaly, Lamia 35100, Greece; 3 Institute of Informatics and Telecommunications, National Center for Scientific Research—“Demokritos”, Athens 15341, Greece

## 1. Background

In recent years, following the tremendous growth of the Web, extremely large amounts of digital multimedia content are being produced every day and are shared online mainly through several newly emerged channels, such as social networks. Moreover, several digital content archives and datasets have become publicly available. Therefore, the field of image-based information retrieval has received a great deal of attention and on a wide range of topics dealing with every aspect of content-handling. When designing and implementing an image-based retrieval system, and considering the continuous growth of digital content, one must deal with several issues such as efficiency, accuracy, scaling, user-friendliness, and impact. The intent of this special issue is to collect the experiences of leading scientists, but also to serve as an assessment tool for people who are new to the world of image-based information retrieval.

## 2. Papers in This Special Issue

We open this special issue with the paper entitled “*Efficient Implementation of Gaussian and Laplacian Kernels for Feature Extraction from IP Fisheye Cameras*” by Konstantinos Delibasis [1]. The paper deals with traditional image processing techniques, such as the Gaussian kernel, its partial derivatives, and the Laplacian kernel, applied at different image scales. The author claims that aforementioned elements play a very important role in image processing and in image-based feature extraction, and, although they have been extensively studied in the case of images acquired by projective cameras, this is not the case for cameras with fisheye lenses. The latter type of cameras is becoming very popular lately, since it exhibits a field of view (FoV) of 180 degrees. The model of fisheye image formation differs substantially from the simple projective transformation, causing straight lines to be imaged as curves. Thus, traditional kernels used for processing images acquired by projective cameras, are not optimal for fisheye images. The work presented in this research paper uses the calibration of the acquiring fisheye camera to define a geodesic metric for distance between pixels in fisheye images and subsequently redefines the Gaussian kernel, its partial derivatives, as well as the Laplacian kernel. Finally, algorithms for applying in the spatial domain these kernels, as well as the Harris corner detector, are proposed, using efficient computational implementations. Comparative results are shown within the paper, in terms of correctness of image processing, efficiency of application for multi-scale processing, as well as salient point extraction. In this manner, the author concludes that the proposed algorithms allow the efficient application of standard processing and analysis techniques of fisheye images, in the spatial domain, once the calibration of the specific camera is available.

We continue with the second paper entitled “*Digital Comics Image Indexing Based on Deep Learning*” prepared by Nhu-Van Nguyen, Christophe Rigaud, and Jean-Christophe Burie [2], which tackles the application domain of digital comics. The authors claim that digitized comics suffer from a limited automatic content understanding which restricts online content search and reading applications. Their study proposes ways to efficiently combine state-of-the-art image analysis methods to encode and index images into an XML-like text file, also known as a content description file; the latter may then be used to automatically split comic book images into sub-images corresponding to panels, easily indexable with relevant information about their respective content. This technique allows advanced search in keywords said by specific comic characters, action, and scene retrieval using natural language processing. The authors get down to panel, balloon, text, comic character and face detection using traditional approaches and breakthrough deep learning models, and also text recognition using an LSTMmodel, coupled together with a decent evaluation on a dataset composed of online library content, as well as a new public dataset.

The third paper of the special issue entitled “*An Ensemble SSL Algorithm for Efficient Chest X-ray Image Classification*”, prepared by Ioannis Livieris, Andreas Kanavos, Vassilis Tampakas, and Panagiotis Pintelas [3] focuses on computer-aided medical diagnosis based on digital chest X-rays. According to the authors, a critical component in this case is the automatic detection of lung abnormalities, since the effective identification at an initial stage constitutes a significant and crucial factor in patients’ treatment. The vigorous advances in computer and digital technologies have ultimately led to the development of large repositories of labeled and unlabeled images. Due to the effort and expense involved in labeling data, training datasets are of a limited size, while in contrast, electronic medical record systems contain a significant number of unlabeled images. Semi-supervised learning algorithms have become a hot topic of research as an alternative to traditional classification methods, exploiting the explicit classification information of labeled data with the knowledge hidden in the unlabeled data for building powerful and effective classifiers. In the herein presented work, authors evaluate the performance of an ensemble semi-supervised learning algorithm for the classification of chest X-rays of tuberculosis. The efficacy of the presented algorithm is demonstrated by several experiments and confirmed by statistical non-parametric tests, illustrating that reliable and robust prediction models could be developed using a few labeled, as well as many unlabeled data.

The fourth paper of the special issue entitled “*Image-Based Surrogates of Socio-Economic Status in Urban Neighborhoods Using Deep Multiple Instance Learning*” is proposed by Christos Diou, Pantelis Lelekas, and Anastasios Delopoulos [4] and tackles a challenging problem by proposing a methodology for automatically computing surrogate variables of socio-economic status (SES) indicators using street images of parked cars and deep multiple instance learning. In principle, evidence-based policy-making requires data about the local population’s SES at detailed geographical level; however, such information is often not available, or is too expensive to acquire. Researchers have proposed solutions to estimate SES indicators by analyzing Google Street View images; however, these methods are also resource-intensive, since they require large volumes of manually labeled training data. The authors’ approach does not require any manually created labels, apart from data already available by statistical authorities, while the entire pipeline for image acquisition, parked car detection, car classification, and surrogate variable computation is fully automated. The proposed surrogate variables are then used in linear regression models to estimate the target SES indicators. They implement and evaluate a model based on the proposed surrogate variable at 30 municipalities of varying SES in Greece. The model has R2 = 0.76 and a correlation coefficient of 0.874 with the true unemployment rate, while it achieves a mean absolute percentage error of 0.089 and mean absolute error of 1.87 on a held-out test set. Similar results are also obtained for other socio-economic indicators, related to education level and occupational prestige. Consequently, the proposed methodology can be used to estimate SES indicators at the local level automatically, using images of parked cars detected via Google Street View, without the need for any manual labeling effort.

Finally, we close our special issue with the paper entitled “*DIRT: The Dacus Image Recognition Toolkit*” by Romanos Kalamatianos, Ioannis Karydis, Dimitris Doukakis, and Markos Avlonitis [5] that provides an overview of modern agriculture; the latter is facing unique challenges in building a sustainable future for food production, in which the reliable detection of plantation threats is of critical importance. The breadth of existing information sources, and their equivalent sensors, can provide a wealth of data which, to be useful, must be transformed into actionable knowledge. Approaches based on Information Communication Technologies (ICT) have been shown to be able to help farmers and related stakeholders make decisions on problems by examining large volumes of data while assessing multiple criteria. In this paper, the authors address the automated identification (and count the instances) of the major threat of olive trees and their fruit, the Bactrocera Oleae (a.k.a. Dacus) based on images of the commonly used McPhail trap’s contents. Accordingly, they introduce the so-called “Dacus Image Recognition Toolkit” (DIRT), i.e., a collection of publicly available data, programming code samples, and web-services focused at supporting research aiming at the management the Dacus, as well as extensive experimentation on the capability of the proposed dataset in identifying Dacuses using deep learning methods. Experimental results indicated performance accuracy (mAP) of 91.52% in identifying Dacuses in trap images featuring various pests. Moreover, the results also indicated a trade-off between image attributes affecting detail, file size and complexity of approaches and mAP performance that can be selectively used to better tackle the needs of each usage scenario.

## 3. Conclusions

In conclusion, we consider that among the actual goals of the current special issue was to bring together updated implementations, trends, and applications that focus on innovative, as well as existing, works on image-based information retrieval. We believe that it managed to demonstrate the broad diversity of state-of-the-art approaches, as well as the dynamics that exist within the different domains of application. We feel that current trends in the broader area of deep learning in computer vision will continue to attract research efforts at least for the next few years. Among them, we feel that the most well-attended ones will be (a) the usage of synthetic data for model training [6,7], for improvement of performance of deep architectures; (b) domain adaptation [8,9,10], where the goal is to learn a model that performs well on some dataset, while it has been trained on another related dataset; (c) transfer learning [11,12], a useful technique when the number of the available training data is limited/insufficient, yet there exists a trained architecture which may be used for the initialization of training; (d) understanding of 3D objects either by using depth images or point clouds [13,14], where the goal is to move a step beyond traditional 2D image tasks.

Last, but not least, we would also like to express our sincere gratitude to all reviewers of this special issue for making it possible and for devoting their valuable time over the reviewing period. Moreover, we would like to thank all authors for providing their high-quality contributions and the entire editorial staff for their valuable comments, suggestions, and support throughout the entire publishing process.

## References

[1] Delibasis K. (2018). Efficient Implementation of Gaussian and Laplacian Kernels for Feature Extraction from IP Fisheye Cameras. J. Imaging.

[2] Nguyen N., Rigaud C., Burie J. (2018). Digital Comics Image Indexing Based on Deep Learning. J. Imaging.

[3] Livieris I., Kanavos A., Tampakas V., Pintelas P. (2018). An Ensemble SSL Algorithm for Efficient Chest X-ray Image Classification. J. Imaging.

[4] Diou C., Lelekas P., Delopoulos A. (2018). Image-Based Surrogates of Socio-Economic Status in Urban Neighborhoods Using Deep Multiple Instance Learning. J. Imaging.

[5] Kalamatianos R., Karydis I., Doukakis D., Avlonitis M. (2018). DIRT: The Dacus Image Recognition Toolkit. J. Imaging.

[6] Tremblay J., Prakash A., Acuna D., Brophy M., Jampani V., Anil C., Birchfield S. Training deep networks with synthetic data: Bridging the reality gap by domain randomization. Proceedings of the IEEE Conference on Computer Vision and Pattern Recognition Workshops.

[7] Saleh F.S., Aliakbarian M.S., Salzmann M., Petersson L., Alvarez J.M. Effective use of synthetic data for urban scene semantic segmentation. Proceedings of the European Conference on Computer Vision.

[8] Pinheiro P.O. Unsupervised domain adaptation with similarity learning. Proceedings of the IEEE Conference on Computer Vision and Pattern Recognition.

[9] Murez Z., Kolouri S., Kriegman D., Ramamoorthi R., Kim K. Image to image translation for domain adaptation. Proceedings of the IEEE Conference on Computer Vision and Pattern Recognition.

[10] Hong W., Wang Z., Yang M., Yuan J. Conditional generative adversarial network for structured domain adaptation. Proceedings of the IEEE Conference on Computer Vision and Pattern Recognition.

[11] Rampasek L., Goldenberg A. (2018). Learning from everyday images enables expert-like diagnosis of retinal diseases. Cell.

[12] Weinshall D., Cohen G., Amir D. (2018). Curriculum learning by transfer learning: Theory and experiments with deep networks. arXiv.

[13] Girdhar R., Gkioxari G., Torresani L., Paluri M., Tran D. Detect-and-track: Efficient pose estimation in videos. Proceedings of the IEEE Conference on Computer Vision and Pattern Recognition.

[14] Zhou Y., Tuzel O. Voxelnet: End-to-end learning for point cloud based 3d object detection. Proceedings of the IEEE Conference on Computer Vision and Pattern Recognition.

